# Planktonic Interference and Biofilm Alliance between Aggregation Substance and Endocarditis- and Biofilm-Associated Pili in Enterococcus faecalis

**DOI:** 10.1128/JB.00361-18

**Published:** 2018-11-26

**Authors:** Irina Afonina, Xin Ni Lim, Rosalind Tan, Kimberly A. Kline

**Affiliations:** a Singapore Centre for Environmental Life Science Engineering, Nanyang Technological University, Singapore, Singapore; b School of Biological Sciences, Nanyang Technological University, Singapore, Singapore; Indiana University Bloomington

**Keywords:** Enterococcus faecalis, horizontal gene transfer, aggregation substance, endocarditis- and biofilm-associated pili, biofilm

## Abstract

Like many bacteria, Enterococcus faecalis encodes a number of adhesins involved in colonization or infection of different niches. Two well-studied E. faecalis adhesins, aggregation substance (AS) and endocarditis- and biofilm-associated pili (Ebp), both contribute to biofilm formation on abiotic surfaces and in endocarditis, suggesting that they may be expressed at the same time. Because different regulatory pathways have been reported for AS and Ebp, here, we examined if they are coexpressed on the same cells and what is the functional impact of coexpression on individual cells and within a population. We found that while Ebp are only expressed on a subset of cells, when Ebp and AS are expressed on the same cells, pili interfere with AS-mediated clumping and impede AS-mediated conjugative plasmid transfer during planktonic growth. However, when the population density increases, horizontal gene transfer rates normalize and are no longer affected by pilus expression. Instead, at higher cell densities during biofilm formation, Ebp and AS differentially contribute to biofilm development and structure, synergizing to promote maximal biofilm formation.

**IMPORTANCE** Most bacteria express multiple adhesins that contribute to surface attachment and colonization. However, the network and relationships between the various adhesins of a single bacterial species are less well understood. Here, we examined two well-characterized adhesins in Enterococcus faecalis, aggregation substance and endocarditis- and biofilm-associated pili, and found that they exhibit distinct functional contributions depending on the growth stage of the bacterial community. Pili interfere with aggregation substance-mediated clumping and plasmid transfer under planktonic conditions, whereas the two adhesins structurally complement one another during biofilm development. This study advances our understanding of how E. faecalis, a ubiquitous member of the human gut microbiome and an opportunistic pathogen, uses multiple surface structures to evolve and thrive.

## Abstract (precis)

Most bacteria express multiple adhesins that contribute to surface attachment and colonization. However, the network and relationships between the various adhesins of a single bacterial species are less well understood. Here, we examined two well-characterized adhesins in Enterococcus faecalis, aggregation substance and endocarditis- and biofilm-associated pili, and found that they exhibit distinct functional contributions depending on the growth stage of the bacterial community. Pili interfere with aggregation substance-mediated clumping and plasmid transfer under planktonic conditions, whereas the two adhesins structurally complement one another during biofilm development. This study advances our understanding of how E. faecalis, a ubiquitous member of the human gut microbiome and an opportunistic pathogen, uses multiple surface structures to evolve and thrive.

## INTRODUCTION

Enterococcus faecalis is a Gram-positive opportunistic pathogen that causes a variety of infections, such as endocarditis, bacteremia, urinary tract infections (UTI), and catheter-associated urinary tract infections (CAUTI) (1–3). Most infections start with bacterial adhesion to a biotic or abiotic surface, and E. faecalis encodes multiple adhesins that facilitate attachment to and colonization of different niches within the host. Sortase enzymes are conserved within Gram-positive bacteria and catalyze the covalent attachment of many adhesins to the cell wall. Sortase substrates can be predicted based on the presence of a conserved sortase A recognition motif, LPXTG (leucine, proline, X [any amino acid], threonine, and glycine), within a canonical cell wall sorting signal (4). E. faecalis strain V583 encodes 41 predicted SrtA substrates (5). Of these putative substrates, 17 are predicted to be microbial surface component recognizing adhesive matrix molecules (MSCRAMM), although only a few, including endocarditis- and biofilm-associated pili (Ebp) and aggregation substance (AS), have been characterized in detail (2, 6–8).

Ebp are composed of 3 subunits, EbpA, EbpB, and EbpC, where EbpC is the major pilus subunit with EbpB at the base, and EbpA is at the tip of the pilus (2, 9). The three subunits are cotranscribed at the *ebpABC* locus and are positively regulated by the transcriptional regulator EbpR, which is encoded upstream of *ebpABC* (10). Polymerized Ebp exist as high-molecular-weight polymers (>200 kDa), and the length of the pilus may reach 10 μm (5). Pili are only expressed on a subset of cells in the population, suggesting that they may be phase variable, and pilus expression can be induced by exposure to serum, glucose, or bicarbonate (2, 11–13). The tip adhesin EbpA mediates attachment to host fibrinogen and collagen and contributes to UTI, CAUTI, and endocarditis (2, 3, 13). Mutations within the N-terminal domain of EbpA prevented Ebp-associated biofilm formation both *in vitro* and *in vivo*, as well as CAUTI in mice (2, 3, 14).

AS is a 137-kDa protein encoded by *prgB* on the pheromone-responsive pCF10 plasmid (15). In the absence of the cCF10 pheromone, the expression of AS and most of the pCF10 plasmid-carried genes is inhibited by the small plasmid-encoded and constitutively expressed peptide iCF10 (16). Alternatively, the expression of AS can be induced by albumin-lipid complexes in the bloodstream that sequester or degrade iCF10, resulting in the activation of autocrine pheromone signaling (17). Scanning electron microscopy experiments demonstrated that AS is only expressed on a subset of cells in a population, even at saturating concentrations of the cCF10 pheromone (18). When expressed, AS contributes to biofilm formation, cellular aggregation required for conjugative plasmid transfer, and increased virulence in endocarditis models (1, 19–21). In addition, AS facilitates the adherence of E. faecalis to renal tubular cells and intestinal epithelial cells, as well as binding to and survival in neutrophils (22).

Most bacteria encode multiple adhesins; however, they are not always expressed at the same time, and this differential expression can arise via cross-regulation. For example, Escherichia coli Pap pili and type 1 fimbriae are cross-regulated, as are flagella and type 4 pili in Pseudomonas aeruginosa (23–25). Despite an increasing number of characterized and predicted adhesins in E. faecalis, we do not know whether or how adhesin expression is coordinated within a population. In this study, we used the well-characterized E. faecalis adhesins Ebp and AS to understand how the two adhesins may be differentially expressed on different cellular subsets, which could give rise to partitioned adhesive functions within a population. While we detected no transcriptional cross-regulation between the two adhesins, we observed rapid expression of AS in pheromone-induced cultures on nearly all cells, with Ebp coexpression on a subset of those cells. Simultaneous expression of Ebp and AS on the same cells prevented AS-mediated clumping required for conjugative plasmid transfer and, consequently, reduced horizontal gene transfer (HGT). Within biofilms, we demonstrate distinct functional contributions of Ebp and AS to biofilm development and structure, working synergistically to promote biofilm formation.

## RESULTS

### AS and Ebp are coexpressed on the same cells after pheromone induction.

**FIG 1:**
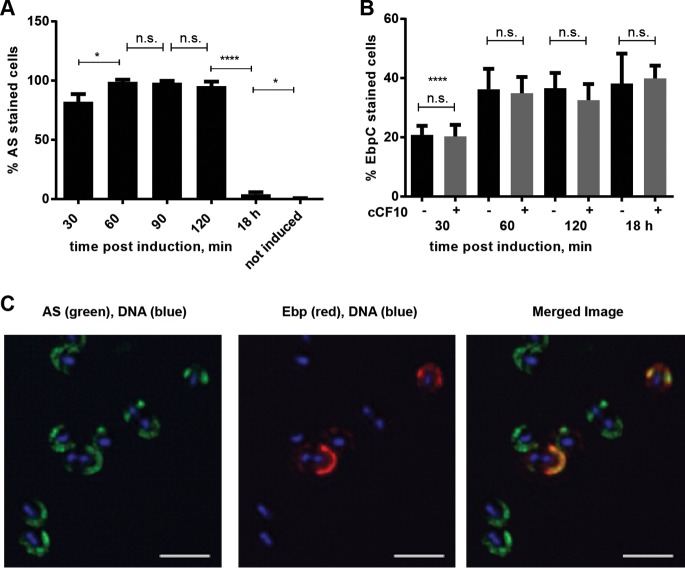
AS and Ebp are coexpressed on the same cells after pheromone induction. (A) IFM was performed with AS antiserum, and the percentage of AS^+^ cells within the population was quantified. (B) IFM with EbpC antiserum was performed on cCF10 pheromone-uninduced (black bars) and -induced (gray bars) OG1RF/pCF10 at the indicated time points, and the percentage of EbpC^+^ cells within the population was quantified. (C) IFM on pheromone-induced cells with Ebp antiserum (red), AS (green), and DNA (blue). Scale bars = 1 μm. For panels A and B, the mean values are shown from 3 independent experiments in which at least 300 cells were counted. Asterisks above the 30-min time point indicate a significant difference for each population compared to all other time points. Error bars represent the standard deviation. Statistical analysis was performed by the unpaired *t* test using GraphPad. *, *P* < 0.05; ****, *P* < 0.0001, n.s., *P* > 0.05.

Previous studies reported that neither AS nor Ebp are expressed on all cells within a population, but both adhesins contribute to biofilm formation (2, 18–20). We hypothesized that Ebp and AS expression may be coordinated within a population such that different population subsets express different adhesin repertoires for optimal colonization, virulence, or biofilm architecture development. To address how the expression of the two adhesins is coordinated, we first quantified the individual expression of AS and Ebp within a population by diluting overnight cultures of E. faecalis strain OG1RF (a rifampin- and fusidic acid-resistant derivative of OG1) into fresh medium containing the cCF10 pheromone (0.12 ng/ml). After 30 min of pheromone exposure, 82% of the cells expressed AS on the cell surface, and this number increased to 95% by 90 min of pheromone exposure (Fig. 1A). The fraction of AS-expressing cells was higher than what we expected based on earlier reports for the same strain in which representative scanning electron micrographs (SEM) of exponentially grown pheromone-induced bacteria showed that only ∼75% of cells expressed AS (18); this difference which may be due to the increased sensitivity of AS detection by immunofluorescence microscopy (IFM) compared to SEM. After 18 h of growth in the presence of cCF10, the percentage of AS-expressing cells in the population dropped significantly to 4% (Fig. 1A). Since AS transcription peaks between 30 and 60 min after the addition of pheromone and returns to uninduced levels by the end of the second hour, the drop in the number of AS-expressing cells is likely due to an accumulation of inhibitor iCF10, along with division and dilution of AS-expressing cells by AS-negative daughter cells (17, 26). Within the same population, Ebp were expressed on only 20 to 40% of cells, and Ebp expression was independent of cCF10 exposure or AS expression (Fig. 1B), indicating that Ebp could be coexpressed with AS on a subset of cells. Interestingly, independent of pheromone exposure, we noticed a significant decrease in the number of piliated cells 30 min after overnight subculturing compared to that in the overnight culture itself. We speculate that in newly replicated early log-phase cells, a sufficient number of pili have yet to be assembled and accumulated on the surface to be detected. Finally, we performed coimmunostaining using AS and EbpC antisera on pheromone-induced cells and observed that the two adhesins were displayed on the same cells and colocalized at the same hemispherical areas of the cell (Fig. 1C; see also Fig. S1 in the supplemental material).

### Ebp interfere with AS-mediated clumping.

**FIG 2:**
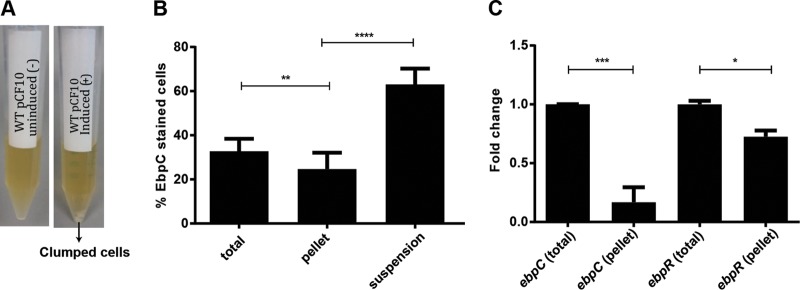
Ebp interfere with AS-mediated clumping. (A) Representative image of OG1RF/pCF10 uninduced (left) and OG1RF/pCF10 induced (right) with pheromone cCF10 (0.12 ng/ml), 2 h after induction. (B) IFM was performed 2 h post-cCF10 induction with EbpC antiserum on the total population, suspension fraction (top 2 ml), and pellet fraction (clumped cells), and the percentage of EbpC^+^ cells was quantified. (C) RT-qPCR on RNA isolated from pheromone-induced cells 2 h post-cCF10 induction. Fold change indicates the change in *ebpC* and *ebpR* transcription in the pellet cells compared to the control (total). Mean values are shown from 3 independent experiments. Error bars represent the standard deviation. Each experiment was performed in triplicate using the ΔΔ*C_T_* method to assess expression changes, with *gyrA* as the reference housekeeping gene. Statistical analysis was performed by unpaired *t* test using GraphPad.*, *P* < 0.05; **, *P* < 0.01, ***, *P* < 0.001; ****, *P* < 0.0001, n.s., *P* > 0.05.

AS was originally described for its association with cellular aggregation, and Ebp have also been shown to contribute to cellular aggregation (27, 28). While we did not observe a difference in Ebp expression between induced AS-expressing and uninduced AS-nonexpressing populations (Fig. 1B), we noticed that not all cells in the cCF10-induced cultures aggregated, but they instead separated into clumped cells that settled at the bottom of the tube as a pellet and cells that remained in suspension. Separation became obvious 1.5 to 2 h after the addition of cCF10 to the cultures (Fig. 2A). We hypothesized that the clumping we observed in pheromone-induced cultures might be both AS and Ebp dependent, where pilus-expressing cells might facilitate AS-mediated clumping. To test this, we manually separated the clumped pellet from the suspended cells, stained them with Ebp antiserum, and performed IFM. Contrary to our hypothesis, we observed fewer Ebp-expressing cells in the clumped pellet and more in the suspension than in the total (mixed) induced culture, where pellets and suspended cells were not separated (Fig. 2B and S2).

We next performed reverse transcription-quantitative PCR (RT-qPCR) on the pellet cells and confirmed that *ebpC* transcript levels were lower in the pellet cells than those in the total population, with a similar transcript reduction of *ebpR*, a positive transcriptional regulator of *ebp* genes (Fig. 2C). Given that Ebp expression levels within the total population were unchanged upon cCF10 pheromone exposure (Fig. 1B), we speculated that pilus-expressing cells may physically segregate to the suspension and be excluded from the aggregated pellet where pilus-mediated steric hindrance interferes with AS-mediated clumping.

### Ebp impede AS-facilitated horizontal gene transfer.

**FIG 3:**
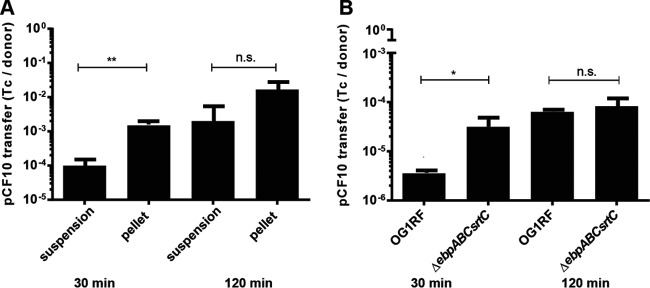
Ebp impede AS-mediated HGT. (A) HGT rates from suspension and pellet cells of donor OG1SS/pCF10 (Str and Tet resistant, Rif sensitive) 2 h post-pheromone induction to plasmid-free Ebp-null recipient OG1RF Δ*ebpABC srtC* (Rif resistant, Str and Tet sensitive). (B) HGT rates from OG1RF/pCF10 and Δ*ebpABC srtC*/pCF10 mutant (both Rif and Tet resistant, Str sensitive) donor cells to plasmid-free recipient OG1X Δ*srtC* (Str resistant, Rif and Tet sensitive). HGT rate is expressed as the number of transconjugants (Tc) per donor cell 30 and 120 min postmating. Error bars represent the standard deviation of the results from 3 independent experiments. Statistical analysis was performed by the unpaired *t* test using GraphPad. *, *P* < 0.05; **, *P* < 0.01, n.s., *P* > 0.05.

Since AS-mediated clumping facilitates conjugative transfer of the pCF10 plasmid to recipient cells (20), nonclumped suspended cells, which express both AS and Ebp, could display a reduced ability to undergo AS-mediated plasmid transfer compared to the pellet cells. To test this, we quantified conjugation frequency using suspension (Ebp^hi^ containing AS [AS^+^]) or pellet (Ebp^lo^ AS^+^) cells using OG1SS/pCF10 as a donor (a streptomycin- and spectinomycin-resistant derivative of OG1 carrying pCF10, which encodes tetracycline resistance). After 2 h of pheromone exposure, we collected the top 2 ml of each culture containing suspended cells and the bottom 1 ml of clumped cells containing the clumped pellet, out of an overall 5-ml culture. We normalized the bacterial cell numbers and mixed them with OG1RF Δ*ebpABC srtC* recipient cells that lack pCF10 (rifampin and fusidic acid resistant). We used Ebp-deficient recipient cells to avoid pilus-mediated interference when incubated with the donor cells. Thirty minutes of coincubation of Ebp^lo^ AS^+^ pellet donor cells and Ebp-null recipient cells yielded approximately 10 times more transconjugants than the Ebp^hi^ AS^+^ suspension donor cell mixture (Fig. 3A), indicating that Ebp interferes with AS-mediated conjugative plasmid transfer.

To further test the hypothesis that pili interfere with AS-mediated HGT, we used OG1RF/pCF10 or OG1RF Δ*ebpABC*/pCF10 (*ebp*-null) as the donor strain and compared pCF10 conjugation rates to that of an OG1X Δ*srtC* recipient strain (streptomycin resistant) that fails to polymerize pili (Fig. 3B). After 30 min of coincubation, the Δ*ebpABC* mutant was approximately 10-fold more efficient in plasmid transfer to OG1X Δ*srtC* than was OG1RF. In another study, similar conjugation experiments performed after 2 h of coculture revealed no differences in HGT for Ebp-null donor strains and OG1RF (20). To address this, we extended our assays from 30 min to 2 h and observed that HGT rates equalized (Fig. 3). This delayed equalization of recovered transconjugants may be due to a second round of transfer of the plasmid from new donors to recipient cells, a switch to “off” piliation from originally “on” piliation donor cells, and/or enhanced cellular density that facilitates cell-to-cell contact and HGT.

### AS-clumped cells mediate microcolony formation and biofilm development.

**FIG 4:**
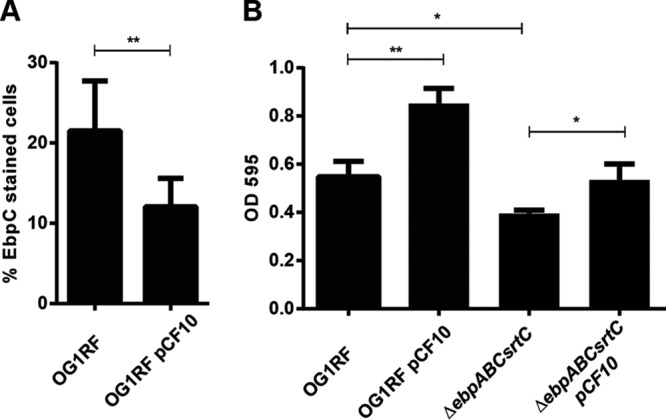
AS contributes to initial attachment and biofilm formation independent of Ebp. (A) IFM with Ebp antiserum was performed on cells attached to the biofilm chamber 2 h after induction. Error bars represent the standard deviation from 3 independent experiments. Statistical analysis was performed by unpaired *t* test using GraphPad. *, *P* < 0.05; **, *P* < 0.01, n.s., *P* > 0.05. (B) CV staining of biofilms formed by OG1RF, OG1RF/pCF10, OG1RF Δ*ebpABC srtC*, and OG1RF Δ*ebpABC srtC*/pCF10 in TSBG medium supplemented with 0.12 ng/ml cCF10 on plastic after 24 h. Error bars represent the standard deviation of the results from 3 independent experiments. Statistical analysis was performed by unpaired *t* test using GraphPad.

**FIG 5:**
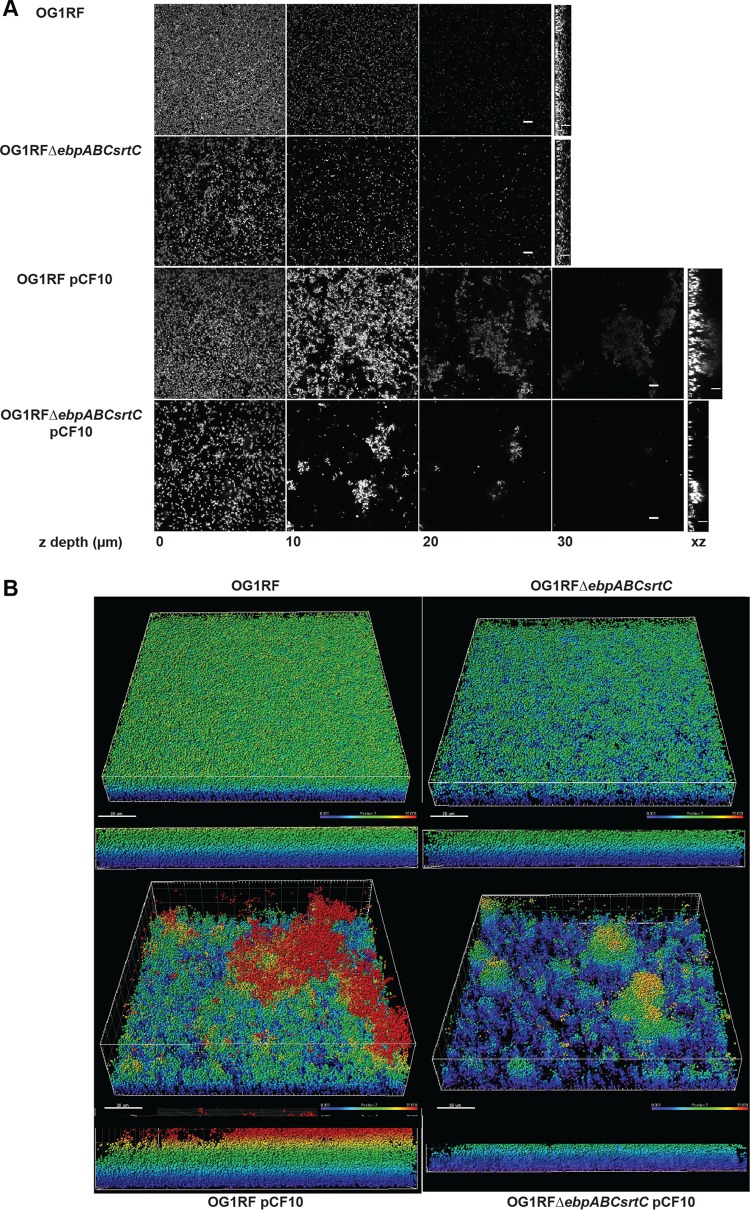
Ebp and AS differentially contribute to biofilm structure and development. Confocal laser scanning microscopy images of 24 h biofilms grown in plastic chambers in 40% TSBG with cCF10 (1.2 μg/ml). Cells were fixed and stained with Hoechst dye. (A) z-stack images represent biofilm depths of 0, 10, 20, and 30 μm, followed by z-view for the same samples. Scale bars are 10 μm. (B) Imaris software modeling of the confocal laser scanning microscopy images from panel A, where every cell is represented as a sphere based on Hoechst staining, and the z-depth is color-coded (0 to 25 μm), where purple is 0 μm and red is 25 μm. Both perspective (top) and side (bottom) views are shown. Scale bars are 20 μm.

While both AS and Ebp contribute to biofilm formation, pheromone induction of OG1RF/pCF10 gives rise to thicker biofilms than with OG1RF without the plasmid (20). Given that AS-expressing OG1RF/pCF10 cells clump after 2 h of pheromone exposure, and those clumps largely exclude Ebp-expressing cells, we explored the possibility that initial biofilm attachment is facilitated by AS-mediated clumping to form microcolonies, while Ebp may be important to enhance biofilm maturation. We therefore compared the number of Ebp-expressing cells between OG1RF and OG1RF/pCF10 that have attached and been incorporated into early biofilms after 2 h of pheromone exposure by scraping the cells from biofilm chambers and performing an indirect immunofluorescence assay (IFA) with an EbpC antibody. Twelve percent of OG1RF/pCF10 and 22% of OG1RF early biofilm cells displayed Ebp on their cell surface, suggesting that if AS is present and induced, it will promote the initial attachment of biofilm without the aid of Ebp-expressing cells (Fig. 4A and S3). To further explore the contributions of Ebp and AS, together and individually, to biofilm development, we assayed biofilm formation by OG1RF (Ebp-expressing [Ebp^+^], AS-nonexpressing [AS^−^]), OG1RF/pCF10 (Ebp^+^ AS^+^), OG1RF Δ*ebpABC srtC* (Ebp-nonexpressing [Ebp^−^], AS^−^) and OG1RF Δ*ebpABC srtC*/pCF10 (Ebp^−^ AS^+^) strains (Fig. 4B). Consistent with previous reports (2), we observed a 30% reduction in biofilm formation by OG1RF Δ*ebpABC srtC* compared to OG1RF and a 54% increase in the biomass of the induced OG1RF/pCF10 strain that expresses both AS and Ebp compared to OG1RF (20) (Fig. 4B). Moreover, we observed similar biofilm biomass for OG1RF and induced OG1RF Δ*ebpABC srtC*/pCF10, suggesting that in the absence of Ebp, AS alone is sufficient to revert the biomass of the e*bpABC*-null strain to wild-type levels (Fig. 4B). To determine how Ebp and AS contribute to biofilm structure and organization, we grew biofilms for 24 h in the presence of cCF10, stained the DNA of all the cells with Hoechst dye, and performed confocal laser scanning microscopy. We observed distinct differences in the biofilm structure and thickness between the strains that express one, both, or neither of the adhesins (Fig. 5). In the absence of AS, OG1RF forms uniform and tightly packed biofilms, while AS-expressing OG1RF/pCF10 cells formed additional 3-dimensional microcolonies, suggesting that AS promotes the formation of structured biofilm (Fig. 5). Moreover, while we observed uniform tightly packed biofilms in Ebp-expressing OG1RF and OG1RF/pCF10, the Δ*ebpABC srtC* and Δ*ebpABC srtC*/pCF10 Ebp-null mutant strains appeared to be sparsely packed (Fig. 5), suggesting that pili are important for tight interactions within biofilms. Furthermore, we observed that AS drives microcolony formation through cellular clumping, since we noticed elevated microcolony clusters only for pCF10-containing AS-expressing strains (Fig. 5B). Finally, pili contributed to the development of thicker biofilms in OG1RF and AS-expressing OG1RF/pCF10 strains than that in their Ebp-null counterparts (Fig. 5B). We therefore conclude that despite accumulating similar overall biofilm biomass (Fig. 4B), OG1RF and OG1RF Δ*ebpABC srtC*/pCF10, displaying opposite repertoires of Ebp and AS, exhibit divergent biofilm development, suggesting that the two adhesins differentially contribute to biofilm structure.

## DISCUSSION

Although most bacteria encode a variety of adhesins for surface attachment and colonization, the number and variety of adhesins expressed in the population and on each cell can vary to maximize adhesive capacities but avoid immune clearance and cross-adhesin interference (23, 29). In E. faecalis V583, 17 of 41 predicted sortase A substrate proteins contain MSCRAMM motifs, and at least 7 are expressed within the human host, as antibodies for them are readily detected in the serum (5). This observation raises questions as to when, to what extent, and how promiscuously each adhesin is expressed within an enterococcal population. In the present study, we addressed the relationship between two of these enterococcal adhesins, Ebp and AS.

We showed that Ebp and AS can be coexpressed on the same cell, but that known regulatory mechanisms for each adhesin (EbpR and cCF10, respectively) do not influence the expression of the other. However, we found that at low cell density during planktonic growth, simultaneous expression of AS and Ebp interferes with AS-mediated intercellular clumping and reduces HGT rates by 10-fold, presumably due to steric interference by protruding Ebp, which could prevent AS from binding to its receptor, lipoteichoic acid (LTA), on neighboring cells (30). Similarly, E. coli type 1 fimbriae can prevent cellular aggregation mediated by self-associating autotransporters (SAAT), suggesting that bulky surface proteins, such as pili, interfere with aggregation and physically mask the function of SAAT-type proteins (31, 32). Therefore, pilus interference with aggregation might be a more general mechanism across bacteria than previously appreciated. Since E. faecalis pili are only expressed on a subset of cells, maintaining a subset of unclumped piliated cells after AS induction may benefit the overall population by limiting the extent to which HGT can occur. Ebp interference may be a general mechanism to protect planktonic cells from abundant plasmid intake, which in turn could contribute to genome stability and increased fitness (33). Notably, we can only detect Ebp-dependent differences in HGT rates at early time points, and the difference disappears 2 h after mating. Similarly, Bhatty et al. reported no difference in plasmid transfer rates from OG1RF or Ebp-null mutant donors after 2 h of mating, but they did not examine earlier time points, as we did in this study (20). Recently, Ebp on OG1RF donor cells was shown to confer a 1.6-fold increase in conjugative transfer of pAMβ1, after 5 h of mating, compared to *ebp*-null donor cells (28). While different plasmids, mating times, and experimental cell densities may explain the differing conclusions about the contribution of Ebp to plasmid transfer in these previous studies, coupled with our data, a model emerges in which Ebp may interfere with HGT at low cell densities but later promote HGT when Ebp-dependent biofilm aggregates start to form, which may instead facilitate plasmid transfer. The reasons for these density-dependent differences remain to be determined.

In addition to its impact on HGT, we also demonstrate that coexpression of Ebp and AS may have unique functional contributions to biofilm development. Previously, it was shown that AS acts cooperatively with environmental DNA (eDNA) to promote biofilm formation, resulting in thicker biofilms (20). Here, we extend that model and propose that AS and Ebp differentially contribute to the structure of the biofilm, where AS-mediated clumps of cells that are not expressing Ebp give rise to three-dimensional (3D) microcolonies that may be enriched for eDNA. Our data suggest that Ebp-expressing cells can then “fill in” the biofilm space, leading to a more densely packed biofilm, as well as contribute to the development of thicker 3D biofilms.

Importantly, we observed similar biomass of OG1RF and an Ebp-null strain that expresses AS, where AS restored biofilm biomass to OG1RF levels even in the absence of pili. However, despite similarities in biomass accumulation, biofilms formed in the presence of only Ebp or AS differed structurally. Taken together, our findings suggest that these adhesins may complement each other during biofilm formation and simultaneously contribute different structural roles during biofilm development.

## MATERIALS AND METHODS

### Bacterial strains and growth conditions.

The Enterococcus faecalis strains used in this study are listed in Table 1. For planktonic growth, strains were grown statically in brain heart infusion (BHI) medium (BD Difco, USA) or on BHI agar (BHI supplemented with 1.5% agarose [1st Base, Singapore]) at 37°C. For biofilm assays, tryptone soy broth (Oxoid, UK) supplemented with 10 mM glucose (TSBG) was used. Antibiotics were used at the following concentrations, where appropriate: tetracycline (Tet), 15 μg/ml; rifampin (Rif), 25 μg/ml; and streptomycin (Str) 500 μg/ml.

**TABLE 1:** Bacterial strains used in this study

Strain	Resistance*^a^*	Phenotype	Reference or source
OG1RF	Rif, Fus, Tet	Ebp^+^ AS^−^	27
OG1RF/pCF10	Rif, Fus, Tet	Ebp^+^ AS^+^	This study
OG1SS/pCF10	Str, Tet	Ebp^+^ AS^+^	38
OG1X Δ*srtC*	Str	Lacks polymerized pili, AS^−^	11
OG1RF Δ*ebpABC srtC*	Rif, Fus	Ebp^−^ AS^−^	9
OG1RF Δ*ebpABC srtC*/pCF10	Rif, Fus, Tet	Ebp^−^ AS^+^	This study

*^a^*Rif, rifampin; Fus, fusidic acid; Tet, tetracycline; Str, streptomycin.

### Strain construction.

To construct OG1RF/pCF10 and OG1RF Δ*ebpABC srtC*/pCF10 strains, we conjugated pCF10 plasmid from OG1SS/pCF10 to OG1RF or OG1RF Δ*ebpABC srtC*, as previously described (27), with the following modifications: overnight cultures of OG1SS/pCF10 were diluted 1:10 in fresh BHI medium and induced with the peptide pheromone cCF10 (LVTLVFV; 1st Base Peptides, Singapore) at a final concentration of 0.12 ng/ml (34) for 1 h, with shaking at 200 rpm until an optical density at 600 nm (OD_600_) of 0.3 was reached. We then added 0.5 ml of the induced culture to 4.5 ml of a mid-log-phase culture of statically grown OG1RF or OG1RF Δ*ebpABC srtC* (overnight cultures were diluted 1:10 in fresh BHI medium and grown for approximately 1.5 h to an OD_600_ of 0.45 to 0.6). The mixed cultures were incubated at 37°C for 30 min with shaking at 200 rpm before plating on BHI agar containing Tet and Rif to select for OG1RF/pCF10 and OG1RF Δ*ebpABC srtC*/pCF10 transconjugants. Transconjugants were validated by PCR for the presence of the pCF10 plasmid-carried gene *prgB* (both strains) and the absence of *ebpC* (only for OG1RF Δ*ebpABC srtC*). The primers used are listed in Table 2.

**TABLE 2:** Primers used in this study

Gene target	Primer name	Sequence (5′ to 3′)	Reference or source
*prgB*	prgB_F	GCCAACAGAAGTTGCACCAG	This study
	prgB_R	CGCATGGCCACCTTTATTCG	This study
*ebpC*	ebpC_F	CGGTCATACCGACGACCAAA	This study
	ebpC_R	TGTCACATCGCCATCGACTT	This study
*gyrA*	gyrA_F	TGTTCGTCGGGATGTGAGTG	10
	gyrA_R	GGTACGCCTTTTTCGATGGC	10
*ebpR*	ebpR_F	GGCAAAAACGTCAACGACCA	This study
	ebpR_R	TCGAGCAAAAGAAGAGCGACT	This study

### HGT assay.

OG1SS/pCF10 donor cells were grown overnight in BHI medium and then diluted to OD_600_ of 0.1 in 5 ml of fresh BHI medium in the presence of 0.12 ng/ml cCF10 peptide. After 2 h of shaking at 200 rpm at 37°C, visible clumps formed on the bottom of the tube. The top 2 ml represented the unclumped (suspension) fraction, and 1 ml from the bottom represented the clumped fraction. EDTA at a final concentration of 0.05 M was added to each fraction and vortexed for 15 s to separate clumped cells (19). Both fractions were normalized to OD_600_ of 1 and washed with phosphate-buffered saline (PBS). Donor cells (from the suspension or clumped fraction) were mixed with recipient OG1RF Δ*ebpABC srtC* cells at a ratio of 1:10 (donor/recipient) and incubated statically at 37°C. After 30 min and 120 min, 1 ml was removed, vortexed for 15 s, serially diluted, and plated on selective plates to quantify donor OG1SS/pCF10 cells on BHI-Str-Tet medium and transconjugants OG1RF Δ*ebpABC srtC*/pCF10 on BHI-Rif-Tet medium. Alternatively, OG1RF/pCF10 and OG1RF Δ*ebpABC srtC* donor cells were grown as described above and were mixed with OG1X Δ*srtC* recipient cells at a ratio of 1:10 (donor/recipient). Conjugation was carried out as described above, and cells were plated on selective plates to quantify donor OG1RF/pCF10 or OG1RF Δ*ebpABC srtC*/pCF10 cells on BHI-Rif-Tet agar and OG1X Δ*srtC*/pCF10 transconjugants on BHI-Str-Tet agar. HGT rates were calculated as the number of transconjugants per donor cell (Tc/donor), as described previously (20).

### Generation of polyclonal antisera.

Recombinant protein fragments were designed, expressed, and purified using the Protein Production Platform (NTU, Singapore), using a technology and workflow from the former biotechnology unit at the Structural Genomics Consortium (Karolinska Institutet, Sweden). Briefly, the AS target (nucleotides 550 to 1100 from *prgB* [NCBI RefSeq accession no. NC_006827.2] [12883.0.16800]) and Ebp target (nucleotides 1 to 627 from NCBI RefSeq accession no. NC_017316.1 [911134.0.913017]) were amplified using a multiconstruct approach to generate 184-amino-acid and 209-amino-acid gene products, respectively, and were cloned in pNIC28-Bsa4 with an N-terminal His tag followed by a TEV protease cleavage site. The resultant plasmid was then transformed to E. coli BL21(DE3), and recombinant protein was expressed following overnight induction with isopropyl β-d-1-thiogalactopyranoside. Cells were lysed, and recombinant protein was purified by immobilized metal-affinity chromatography (IMAC) using the His tag, followed by size-exclusion chromatography. The purity of the recombinant protein was assessed by SDS-PAGE and the mass verified by mass spectrometry. Polyclonal antisera were generated commercially (SABio, Singapore) by immunization of rabbits or guinea pigs with purified recombinant AS or EbpC, respectively. The specificities of the immune sera were confirmed by the absence of signal on Western blots of whole-cell lysates from OG1RF Δ*ebpABC srtC* or a pCF10-free/uninduced strain (Fig. S1).

### Immunofluorescence microscopy.

To image AS and Ebp on planktonically grown E. faecalis, cells were normalized to an OD_600_ of 1.0, washed once with PBS, and then fixed in 4% paraformaldehyde (PFA) for 20 min at 4°C. Cells were again washed, diluted in PBS, and spread on poly-l-lysine-precoated slides (Polysciences, Inc., USA). The slides were then blocked with 2% bovine serum albumin (BSA) in PBS and incubated at room temperature (RT) for 20 min. After blocking, the slides were washed with PBS, and 20 μl of primary antibody (guinea pig anti-Ebp serum and mouse anti-AS serum) was then added to the slides, followed by incubation at 4°C overnight. The next day, the slides were washed 3 times with PBS, and 20 μl of fluorescence conjugated secondary antibody (Alexa Fluor 568–goat anti-guinea pig antibody for Ebp and Alexa Fluor 488–goat anti-rabbit antibody for AS [Invitrogen, Inc., USA]) was added and incubated in the dark for 1 h at RT. Slides were then washed, mounted with VectaShield mounting medium (Vector Laboratories, Inc., USA), and visualized on an inverted epifluorescence microscope (Axio Observer Z1; Zeiss, Germany) or Zeiss Elyra PS.1 for colocalization studies. The percentages of EbpC- and AS-stained cells were determined by comparing total number of cells viewed under phase contrast to the number of stained cells on the merged fluorescent/phase image.

### RNA extraction and RT-qPCR.

Bacteria were harvested at the indicated time points by centrifugation at 10,000 rpm for 1 min. RNA was isolated using the UltraClean microbial RNA isolation kit (Mo Bio Laboratories, Inc., USA) according to the manufacturer's protocol. DNase treatment was performed using the Turbo DNA-free kit (Ambion, USA), according to the protocol. RNA concentration and DNA contamination were assessed using a Qubit 2.0 fluorometer (Invitrogen, USA). The integrity of the RNA was determined using the TapeStation according to the manufacturer's protocol (Agilent Technologies, Inc., USA). RNA with RNA integrity number (RIN) values above 7.5 and DNA contamination at <5% was used for RT-qPCR. Equivalent amounts of RNA were converted to cDNA using the SuperScript III first-strand synthesis SuperMix kit, according to the manufacturer's protocol (Invitrogen). Following cDNA synthesis, RT-qPCR was performed using 2× SYBR FAST qPCR universal MasterMix kit, according to the manufacturer's protocol (Kapa Biosystems, USA). The gyrase A housekeeping gene (*gyrA*) was used as an endogenous control in this study, as described previously (10, 35). The primers used for amplification of *gyrA*, *ebpC*, and *ebpR* are listed in Table 2. The ΔΔ*C_T_* method was used to quantify gene expression differences (36).

### Crystal violet biofilm assay.

Crystal violet biofilm assays were performed in a 96-well plate (Thermo Fisher, USA), as described previously (37), with the following modifications. Overnight cultures were normalized to an OD_600_ of 0.7, washed, and resuspended in 1 ml of PBS. Two hundred microliters of the normalized culture was added to 5 ml of TSBG with or without 0.12 ng/ml cCF10. Two hundred microliters of vortexed cells was seeded into 96-well plates in triplicate and grown at 37°C for 24 h. The wells were then washed 2 times with PBS, stained with 0.1% crystal violet solution (Sigma-Aldrich, Germany), and incubated for 30 min at 4°C. The wells were then washed three times with PBS and blot dried. Two hundred microliters of an ethanol-acetone (80:20) solution was added to each well to solubilize the crystal violet and incubated for 45 min at RT. After incubation, OD_595_ readings were taken using a spectrophotometer (UVmini-1240; Shimadzu, Japan).

### Confocal laser scanning microscopy and 3D modeling.

Bacteria were grown and normalized as described above for a crystal violet biofilm assay. Two hundred microliters from a suspension of 10^5^ CFU/ml in 40% TSBG plus 1.2 ng/ml cCF10 were seeded to 8-well chamber slides (Ibidi, Germany) and incubated at 37°C. After 24 h, supernatants were removed, and the remaining cells were fixed with 4% PFA for 20 min at 4°C. The biofilms were then washed once with PBS, and DNA was stained with Hoechst 33342 dye (Thermo Fisher, USA) at a final concentration of 1 μg/ml for 20 min at RT in the dark. We tried both 0.12 and 1.2 ng/ml pheromone for the biofilm assay, and while we observed similar clumping for pCF10-carrying strains, the biofilms grown in 0.12 ng/ml cCF10 were more delicate, and most cells and clumps lifted easily prior to PFA staining. Therefore, for consistency in handling, we carried out biofilm experiments with 1.2 ng/ml pheromone. Fixed and stained biofilms were imaged directly from the chambers on an LSM780 confocal microscope (Zeiss, Germany) at ×60 magnification with a 405-nm laser. z-stacks were collected through entire biofilm thickness every 0.42 nm before the signal loss. At least 5 z-stacks per chamber were collected at different locations. The Fiji software was used to create biofilm montage representations at increments of 5 nm. Three-dimensional reconstruction and modeling were performed using the Imaris software (Bitplane, USA), where every cell was represented as 0.5-nm sphere based on DNA staining, and the z-depth was color-coded.

## Supplementary Material

Supplemental file 1
