# Supplementary material for: Planktonic Interference and Biofilm Alliance between Aggregation Substance and Endocarditis- and Biofilm-Associated Pili in Enterococcus faecalis
Source: J Bacteriol. 2018 Nov 26;200(24):e00361-18. doi: 10.1128/JB.00361-18 (PMC6256026; doi:10.1128/JB.00361-18)

Figure S1. Ebp and AS are co-expressed on the same cells.

(Top) IFM images of OG1RF pCF10 induced with cCF10 (0.12 ng/ml) and stained with Ebp and AS antibodies. Phase contrast, individual, and merged channels are shown. Boxed cells are magnified in the top right corner. Scale bar is 5 µm. (Middle and bottom) control IFM images for antibody specificity, where EbpC antibody does not bind to Ebp null cells (OG1RFΔ*ebpABCsrtC*) and AS does not stain uninduced OG1RF pCF10. Phase contrast, individual, and merged channels are shown. Scale bar is 10 µm.

Figure S2. Ebp expressing cells are enriched in the suspension and reduced in the pellet fraction.

Representative IFM images of cCF10 (0.12 ng/ml) induced OG1RF pCF10 suspended, pellet and total (mixed) cells stained with EbpC antibody. Phase contrast, individual, and merged channels are shown. Scale bar is 10 µm.

Figure S3. AS-expressing cells facilitate initial attachment independent of Ebp.

Representative IFM images of cCF10 (1.2 ng/ml) induced OG1RF and OG1RF pCF10 cells attached to the biofilm chamber 2 hours after induction, stained with EbpC antiserum. Phase contrast, individual, and merged channels are shown. Scale bar is 10 µm.

Figure S1.

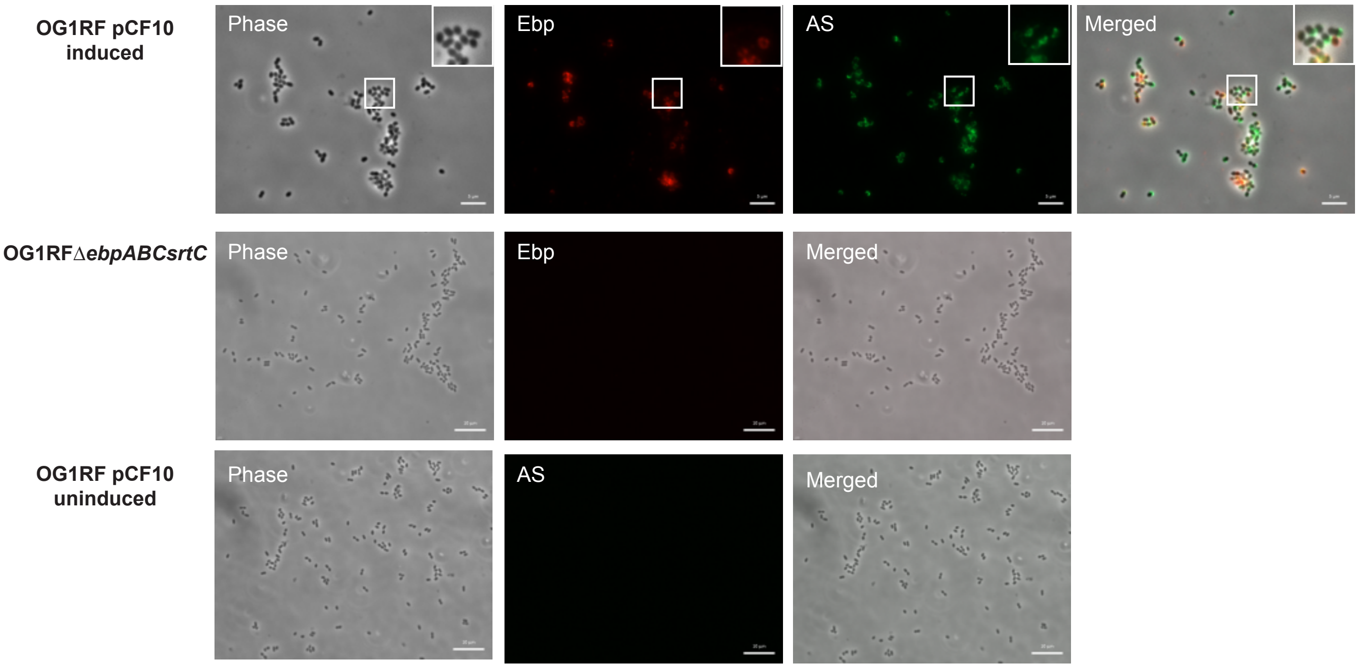

Figure S2.

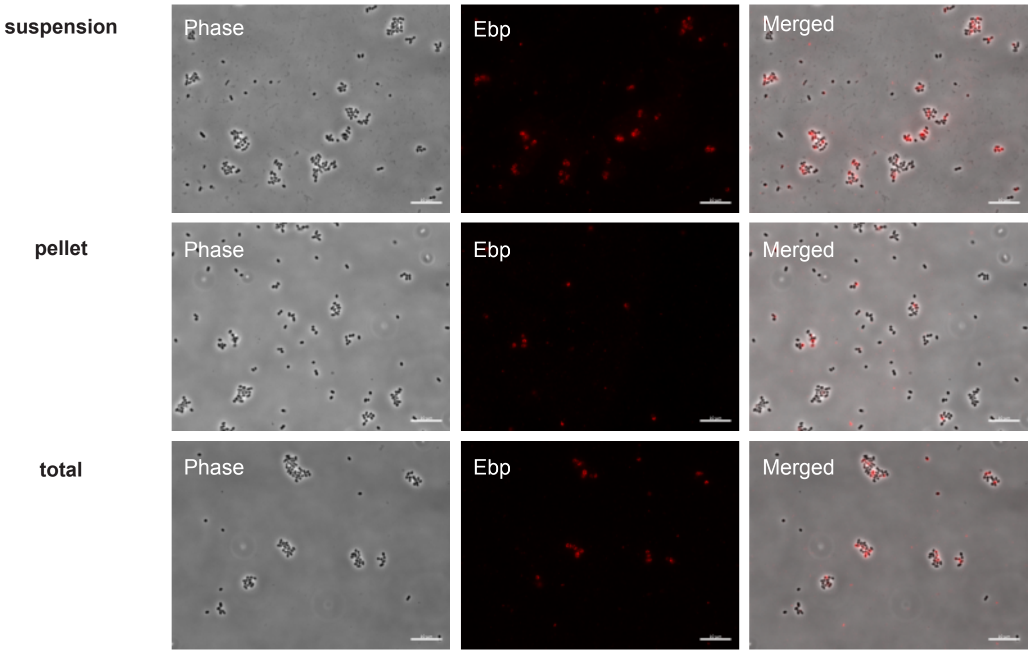

Figure S3.

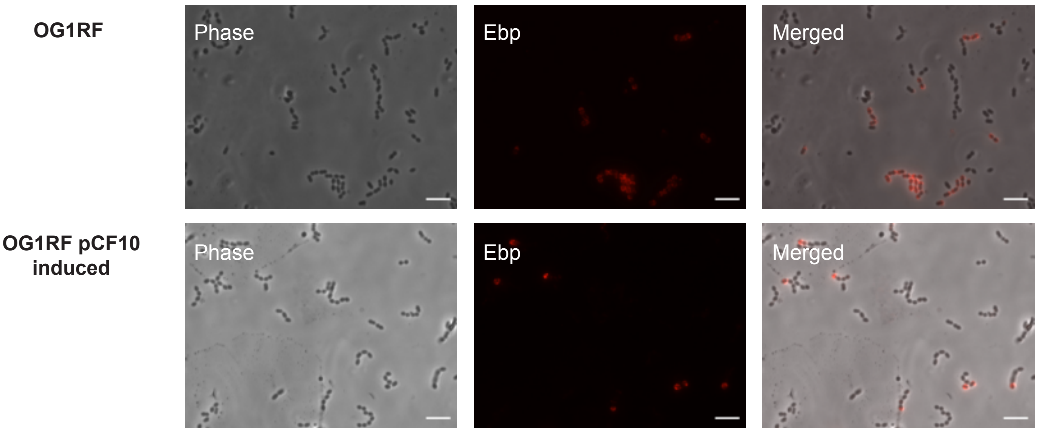

Supplement: Supplemental file 1 [file zjb999094928s1.pdf]
